# Establishment of a novel probe-based RT-qPCR approach for detection and quantification of tight junctions reveals age-related changes in the gut barriers of broiler chickens

**DOI:** 10.1371/journal.pone.0248165

**Published:** 2021-03-05

**Authors:** J. Sophia von Buchholz, Ivana Bilic, Jörg R. Aschenbach, Michael Hess, Taniya Mitra, Wageha A. Awad

**Affiliations:** 1 Department for Farm Animals and Veterinary Public Health, Clinic for Poultry and Fish Medicine, University of Veterinary Medicine, Vienna, Austria; 2 Department of Veterinary Medicine, Institute of Veterinary Physiology, Freie Universität Berlin, Berlin, Germany; Fukushima Medical University, JAPAN

## Abstract

Tight junctions (TJs) play a dominant role in gut barrier formation, therefore, resolving the structures of TJs in any animal species is crucial but of major importance in fast growing broilers. They are regulated in molecular composition, ultrastructure and function by intracellular proteins and the cytoskeleton. TJ proteins are classified according to their function into barrier-forming, scaffolding and pore-forming types with deductible consequences for permeability. In spite of their importance for gut health and its integrity limited studies have investigated the TJs in chickens, including the comprehensive evaluation of TJs molecular composition and function in the chicken gut. In the actual study sequence-specific probes to target different TJ genes (claudin 1, 3, 5, 7, 10, 19, zonula occludens 1 (ZO1), occludin (OCLN) and tricellulin (MD2)) were designed and probe-based RT-qPCRs were newly developed. Claudin (CLDN) 1, 5, ZO1 and CLDN 3, 7, MD2 were engulfed in multiplex RT-qPCRs, minimizing the number of separate reactions and enabling robust testing of many samples. All RT-qPCRs were standardized for chicken jejunum and caecum samples, which enabled specific detection and quantification of the gene expression. Furthermore, the newly established protocols were used to investigate the age developmental changes in the TJs of broiler chickens from 1–35 days of age in the same organ samples. Results revealed a significant increase in mRNA expression between 14 and 21days of age of all tested TJs in jejunum. However, in caecum, mRNA expression of some TJs decreased after 1 day of age whereas some TJs mRNA remained constant till 35 days of age. Taken together, determining the segment-specific changes in the expression of TJ- proteins by RT-qPCR provides a deeper understanding of the molecular mechanisms underpinning pathophysiological changes in the gut of broiler chickens with various etiologies.

## Introduction

Intestinal epithelial cells are tightly connected by intercellular junctional complexes that regulate the passage of ions and molecules through the paracellular pathway and are crucial for the integrity of the epithelial barrier. Tight junctions (TJs) are a complex of trans-membrane proteins, that attach polarized cells to neighbouring cells at the apical region of their lateral membranes [1,2]. The composition of TJ protein clusters plays an important role in maintaining the paracellular barrier by forming channels that allow or restrict permeation of fluids, electrolytes and macromolecules as well as of luminal microorganisms and their secreted products between cells, resulting in epithelial surfaces of different tightness [3]. Consequently, tight junctions play a crucial part in the physiological function of epithelial cells. The degree of sealing of tight junctions varies according to external stimuli along with physiological and pathological conditions [4]. Depending on their localization, two different types of TJ can be distinguished: i) the bi-cellular tight junction at cell-cell contacts of two neighbouring cells and ii) the tri-cellular tight junction at the meeting point of three cells, which leads to the formation of a paracellular tube. Additionally, TJ proteins are commonly classified according to their function into barrier-forming, scaffolding and pore-forming types, altogether with various consequences on permeability. Different *in vivo* and *in vitro* studies report that tight junctions are crucial in order to maintain homeostasis. A loss of TJs’ integrity enables an easy paracellular migration of pathogens as well as the uncontrolled diffusion of toxins from the gut mucosa into the whole body [5–8].

The tight junctions are mainly composed of claudins (CLDN), occludin (OCLN), tricellulin (MD2), TJ-associated zonula occludens proteins and junction-adhesion molecules (JAM) [1,9]. The claudin family is a key component of tight junctions and contains at least 27 members. Most claudins (e.g. CLDN1, CLDN3, and CLDN5) increase epithelial resistance by decreasing cation permeability and are therefore categorized as barrier-forming claudins. Others create cation-selective (e.g. CLDN2) or anion-selective (e.g. CLDN17) pores, consequently increasing permeability and can be considered pore-forming claudins. A third group of claudins (e.g. CLDN7 and CLDN16) has ambiguous functions and may increase or decrease permeability [10–12].

Occludin (OCLN) is a TJ protein that is able to shift to various paracellular locations and is therefore able to alter the epithelial permeability [13]. Movement of occludin from the tight junction into cytoplasmic vesicles occurs frequently during barrier function loss and has been shown to be due to multiple stimuli such as oxidative stress and inflammation [14,15]. Zonula occludens-1 (ZO-1) was the first identified TJ-associated protein interacting with several other TJ proteins and can be categorized as scaffolding protein [16,17]. It localizes to the cytoplasmic surface of the cell membrane interacting with most transmembrane TJ proteins [17–19]. So far, the TJ protein tricellulin (MD2) has not yet been recognized in chickens. However, studies in other species have shown that MD2 plays an important role in sealing the tricellular junctions of three neighbouring cells and is somehow crucial for the TJ barriers against macromolecular passage [8]. In poultry, expression patterns of a large number of claudins has been analyzed during chick embryogenesis [20–22]. Developmental analysis of the chickens small intestine after hatching has been performed for CLDN1, CLDN3, CLDN4, CLDN5, CLDN16 as well as the TJ proteins OCLN and ZO-1 and ZO-2 [23,24]. Recently, it was revealed in many studies that the integrity of the chickens intestine has been disrupted by alteration in the mRNA expression of ZO-1, ZO-2, OCLN, CLDN1, CLDN3, CLDN4 and CLDN5 [25–28].

Reverse transcriptase quantitative polymerase chain reaction (RT-qPCR), as an accurate, rapid, sensitive and affordable method, remains one of the genomic methods of choice for the quantification of specific mRNA sequences [29]. It has arisen as a robust and widely used methodology for biological investigation as it can detect and quantify very small amounts of specific nucleic acid sequences. As a research tool, one of the major applications of this technology is the accurate and rapid assessment of physiology, pathophysiology, and developmental changes in gene expression. This method can be applied to measure the expression of target genes in a variety of samples from tissues, blood and cultured cells originating from humans, animals, plants and microorganisms [30,31]. Its principle is based on the comparison of the samples’ starting quantity and of its amplification during the PCR process. Thereby, the quantification cycle (Cq) specifies the first detection time point of the samples´ amplification [31]. RT-qPCR can either be performed with a non-specific DNA binding dye such as SYBR-Green or by using a fluorescence labeled internal DNA probe. Internal DNA probes detect only a target specific DNA sequence which increases the specificity in the presence of other unspecific PCR products or primer-dimers. The ‘TaqMan’ probe is seemingly the most popular among the internal probes. It is based on DNA–based probe with a fluorophore at one end and a quencher of the fluorescence at the opposite end of the probe. The close proximity of the fluorophore and the quencher prevents the fluorescence. The hydrolysis of the fluorescently labelled probe by Taq DNA polymerase, ultimately causes the release of the fluorophore which fluorescence can be detected after excitation with a laser [30–32].

So far, existing expression patterns of tight junctions in poultry samples were not assessed by applying a fluorescent labelled probe. Therefore, the aim of this study was to develop a standardized method for the specific detection and quantification of a larger set of chicken TJ mRNAs and to investigate the age-dependent developmental changes in the TJs expression.

## Materials and methods

### Ethics statement

The animal trial was approved by the institutional ethics committee of the University of Veterinary Medicine and the Ministry of Research and Science under the license number GZ 68.205/0179-V/3b/2018.

### Primer and probe design

Primers and probes targeting different TJ proteins were developed by the GenScript Biotech Real-time PCR (TaqMan) Primer Design Tool with default settings using the sequences available in the NCBI database (Table 1). During primer and probe design, attention was paid to preferably use an intron-spanning region. The specificity of all primers and probes was controlled by NCBI BLASTN algorithm to ensure they were unlikely to amplify other genes. Reference genes RPL13 and TBP were chosen according to a previous study [33].

**Table 1:** Information on selected tight junction genes used for RT-qPCR.

Gene symbol	Full name	Predicted function	RefSeq number for chicken	Primer and probe sequences (F-forward primer; R-reverse primer; P-probe)	F-&R-Primer concentration (nM)
CLDN1	Claudin 1	Barrier forming	NM_001013611	F: AGCCTGGCTTAACTGAGTGT	200
				R: TGCTAGCCGTTGTAGCTGTA	
				P: FAM-TGGCCAAGGGCTGTAACACAATCATCT-BHQ1	
CLDN5	Claudin 5	Barrier forming	NM_204201	F: GCAGGCAAATAACTGCTTGGA	400
				R: AAAGTCTCAAAGGCGCACAG	
				P: CY5-TTTGGGCCCTCAAAGCGCTGA-BHQ2	
ZO1	Zonula occludens 1	Scaffolding function	XM_413773	F: CGTTCACGATCTCCTGAC	200
				R: CTGGTTTAGTTACCCTTTCATC	
				P: ROX-TCAGAGCCTTCAGACCATTCCAG-BHQ2	
CLDN10 V1	Claudin 10 transcript variant 1	Pore forming	NM_001277767.1	F: TCCAACTGCAAGGACTTCCC	400
				R: GCCAAAGAAACCCAGACAGAC	
				P: HEX-CTGGCTCTCGACGGTTACATCCAAGCCT-BHQ2	
CLDN19	Claudin 19	Pore forming	NM_148960	F: GGGCTCTCATGGTTATTTC	400
				R: CAACCTTGGTGCATTTCA	
				P: FAM-CACGACGCTCACGATGATGC-BHQ1	
OCLN	Occludin	Scaffolding and barrier forming	NM_205128	F: GTCTGTGGGTTCCTCATC	400
				R: CCAGTAGATGTTGGCTTTG	
				P: CY5-TCATCCTGCTCTGCCTCATCTG-BHQ2	
MARVELD2	Marvel domain containing 2	Considered to form barrier	XM_424965.6	F: AGGCCACATTCCCAAACCTA	100
				R: ACTGATCGTTGAACACTGCT	
				P: CY5-ACCGGTCCCGCATTTCATTTGTTTGA-BHQ2	
CLDN3	Claudin 3	Barrier forming	NM_204202	F: AAGGCCAAGATCACCATC	100
				R: CAGCGGGTTGTAGAAATC	
				P: ROX-CGGCGTCATCTTCCTGCTCT-BHQ2	
CLDN7	Claudin 7 transcript variant X1 and X2	Ambiguous function	XM_025146512.1 XM_025146513.1	F: CATCGCCTGCTCCTGGTA	200
				R: GCCCAACTCGTACTTGAGGT	
				P: FAM-CGCCCACCGCGTCATCAGCG-BHQ1	

### Sample preparation

One-day-old broiler chickens (males and females) were obtained from a commercial hatchery (Ross-308, Geflügelhof Schulz, Graz, Austria). The birds were housed on wood shavings and were given ad libitum access to water and a commercial diet. No further additives were used for feeding. For RT-qPCR standardization samples of jejunum, representing the small intestine, and caecum, representing the large intestine, were collected from three different broiler chickens at 21 days post hatching (DPH). For the analysis of age associated changes, five broiler chickens independent of their sex were sampled every week starting on day of hatch, 7, 14, 21, 28, and 35 DPH. Immediately after euthanasia of chickens, jejunum and caecum were collected and rinsed with ice-cold phosphate buffered saline at different time points. During postmortem examination, no gross pathological lesions could be observed in any of the birds selected for sampling. Thereafter, intestinal segments were immersed in the RNA stabilization reagent RNA-later (Qiagen, Hilden, Germany) and frozen at -80°C until further use.

### Total RNA extraction and analysis for purity and integrity

After thawing, approximately 25 mg of intestinal sections including the epithelial layer were collected in 2ml Eppendorf tubes on ice and homogenized twice for 2min at 30Hz using Tissue lyser (Qiagen, Hilden, Germany) Immediately after homogenization, total RNA was extracted using the RNeasy^®^ Plus Mini Kit (Qiagen, Hilden, Germany) according to manufacturer’s instructions. Extracted RNA underwent a clean-up using DNaseI (Qiagen) to eliminate genomic DNA. The extracted RNA was stored at -80°C until further use.

The concentration and purity of RNA were measured using A260/280 and A260/230 ratio by Nanodrop2000 spectrophotometer (Thermo Fisher Scientific, Vienna, Austria). All RNA samples used for RT-qPCR were within the range of 1.5 and 2.3 ratio. The nucleic acid purity was equal or above 2. For the assessment of RNA integrity (RIN), the samples were diluted to 300ng/μl with RNase-free water and then analyzed by Bioanalyzer_2100 (Agilent Technologies, Waldbronn, Germany), using RNA 6000 Nano Kit (Agilent Technologies) according to manufacturer’s instructions. For all samples, the RNA Integrity Number (RIN) was determined. The RIN value of all samples ranged between 8 and 10, indicating their integrity.

### RT-qPCR

TaqMan probe chemistry based one step RT-qPCR has been performed by using Brilliant III Ultra-Fast QRT-PCR master mix kit (Agilent Technologies, Waldbronn, Germany), AriaMx real-time PCR system (Agilent Technologies) and the Agilent AriaMx v1.7 software (Agilent Technologies). The thermal cycle profile was as follows: reverse transcription phase for 10 minutes at 50°C followed by the hot start phase at 95°C for 3minutes and 40 cycles of amplification at 95°C for 5s and 60°C for 30s. To determine the optimal primer concentration, concentrations of 100-400nM for primers and 50-200nM for probes were tested beginning with 5dilution points of RNA with a 10-fold serial dilution (100, 10, 1, 0.1, 0.01 ng) for occludin in jejunum and caecum, whereas 5-fold serial dilutions of RNA (100, 40, 8, 0.16, 0.032 ng) were applied for the remaining TJ proteins (Tables 2–5). For those TJ proteins, which did not give signal for 5 dilution points, 3 dilution points were considered sufficient. Each biological sample for RT-qPCR standardization and analysis of age associated changes were run twice as technical replicates with 2μl of RNA. Additionally, each sample was tested for a possible genomic DNA contamination and ran in duplicate without reverse transcriptase for each gene of interest. Furthermore, each run included two wells with no template control (NTC). The present investigation complies the MIQE guidelines [34].

**Table 2:** Singleplex standardization for jejunum samples.

Biological replicate	Gene symbol	Serial dilution (ng/μl)	Slope	Efficiency	R^2^
1	CLDN19	40;8;0.16	-3.239	103.6	0.988
2			-3.209	105.0	0.986
3			-3.229	104.0	0.996
1	OCLN	50;5;0.5	-3.245	103.3	0.998
2			-3.168	106.9	0.997
3			-3.110	109.7	0.999
1	CLDN10	40;8;0.16	-3.214	104.7	0.996
2			-3.120	109.2	0.997
3			-3.355	98.6	0.994

**Table 3:** Singleplex standardization for caecum samples.

Biological replicate	Gene symbol	Serial dilution (ng/μl)	Slope	Efficiency	R^2^
1	CLDN19	40;8;0.16	-3.232	103.9	0.99
2			-3.168	106.9	0.997
3			-3.118	109.3	0.992
1	OCLN	50;5;0.5	-3.186	106.0	0.987
2			-3.239	103.6	0.997
3			-3.531	92.0	0.997
1	CLDN10	40;8;0.16	-3.252	103.0	1.000
2			-3.259	102.7	1.000
3			-3.390	97.2	0.985

**Table 4:** Multiplex standardization for jejunum samples.

Biological replicate	Gene symbol	Serial dilution (ng/μl)	Slope	Efficiency	R^2^
1	CLDN7	100;40;8	-3.236	103.7	0.995
	MD2		-3.581	90.2	1.000
	CLDN3		-3.118	109.3	1.000
2	CLDN7		-3.293	101.2	0.995
	MD2		-3.262	102.6	0.996
	CLDN3		-3.131	108.7	0.991
3	CLDN7		-3.197	105.5	0.989
	MD2		-3.459	94.6	0.998
	CLDN3		-3.180	106.3	0.987
1	CLDN1	100;40;8	-3.336	99.4	0.990
	ZO1		-3.171	106.7	0.985
	CLDN5		-3.159	107.3	0.999
2	CLDN1		-3.163	107.1	0.992
	ZO1		-3.274	102	0.993
	CLDN5		-3.131	108.7	0.999
3	CLDN1		-3.484	93.7	0.995
	ZO1		-3.215	104.7	0.994
	CLDN5		-3.187	105.9	0.993

**Table 5:** Multiplex standardization for caecum samples.

Biological replicate	Gene symbol	Serial dilution (ng/μl)	Slope	Efficiency	R^2^
1	CLDN7	100;40;8	-3.184	106.1	0.996
	MD2		-3.324	99.9	0.998
	CLDN3		-3.247	103.2	0.986
2	CLDN7		-3.169	106.8	0.999
	MD2		-3.284	101.6	0.999
	CLDN3		-3.340	99.3	0.995
3	CLDN7		-3.223	104.3	0.993
	MD2		-3.275	102.0	0.993
	CLDN3		-3.251	103.1	0.993
1	CLDN1	100;40;8	-3.310	100.5	0.991
	ZO1		-3.144	108.0	0.992
	CLDN5		-3.103	110.0	0.999
2	CLDN1		-3.583	90.2	0.995
	ZO1		-3.122	109.1	0.998
	CLDN5		-3.138	108.3	0.997
3	CLDN1		-3.478	93.9	0.986
	ZO1		-3.458	94.6	0.989
	CLDN5		-3.245	103.3	0.997

For sample normalization reference genes RPL13 and TBP were chosen and run in duplex. The following formula was used according to Chrzastek *et al*.(2014) [35] for Cq normalization and adjusted to our RT-qPCR system: C_q_ + (N´_q_−C´_q_) x S/S´, where N´_q_ is the mean C_q_ for the target genes’ RNA in the sample and S and S´ are the average slopes of the regressions of the standard plots for the 3 test mRNA and the target genes’ RNA, respectively [35]. Furthermore, selecting those particular TJs was decided according to the TJ proteins that have been previously expressed in the intestine (also in mammals) in many studies, as well as the possibility to standardize a well-functioning probe based RT-qPCR for the mentioned TJs.

## Statistical analysis

Data are presented as means with Standard Error of the Means (SEM). Following tests for normality (Kolmogorov-Smirnov’s test), statistical analysis of TJs expression was evaluated for significant differences between different age using one-way ANOVA and Duncan’s multiple range test. Differences were considered significant at a level of P ≤ 0.05. All tests were performed using IBM SPSS statistics 24, SPSS software (Chicago, IL, USA).

## Results

### Standardization of RT-qPCR targeting genes encoding tight junction proteins

**Fig 1:**
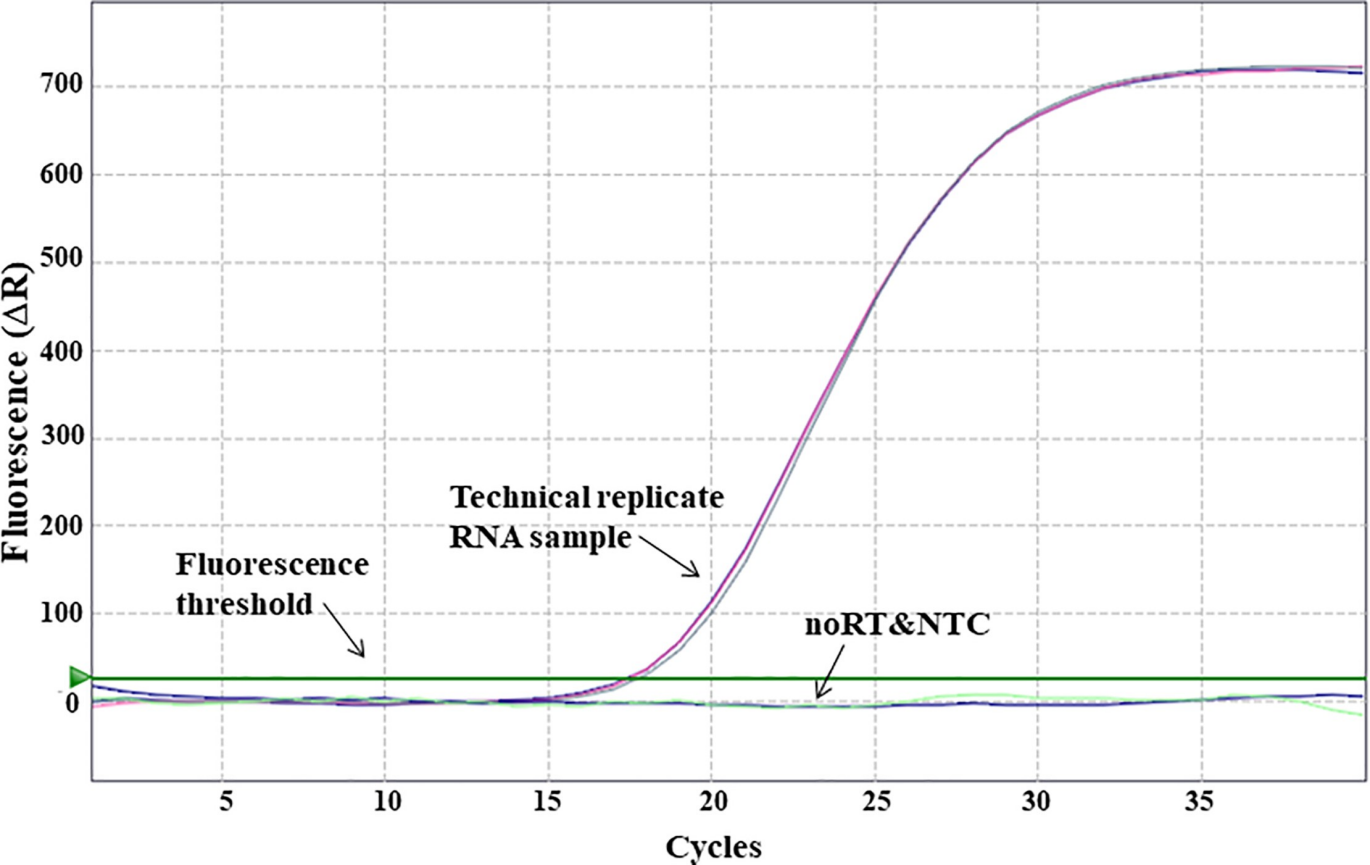
Exemplary amplification curve for standardized target genes: Threshold is set low enough to be within the exponential growth range but above the baseline. No background signals are visible above the baseline, and neither are samples run with no reverse transcriptase noRT or no template control NTC. Running samples were performed in triplicate to control technical variability.

**Fig 2:**
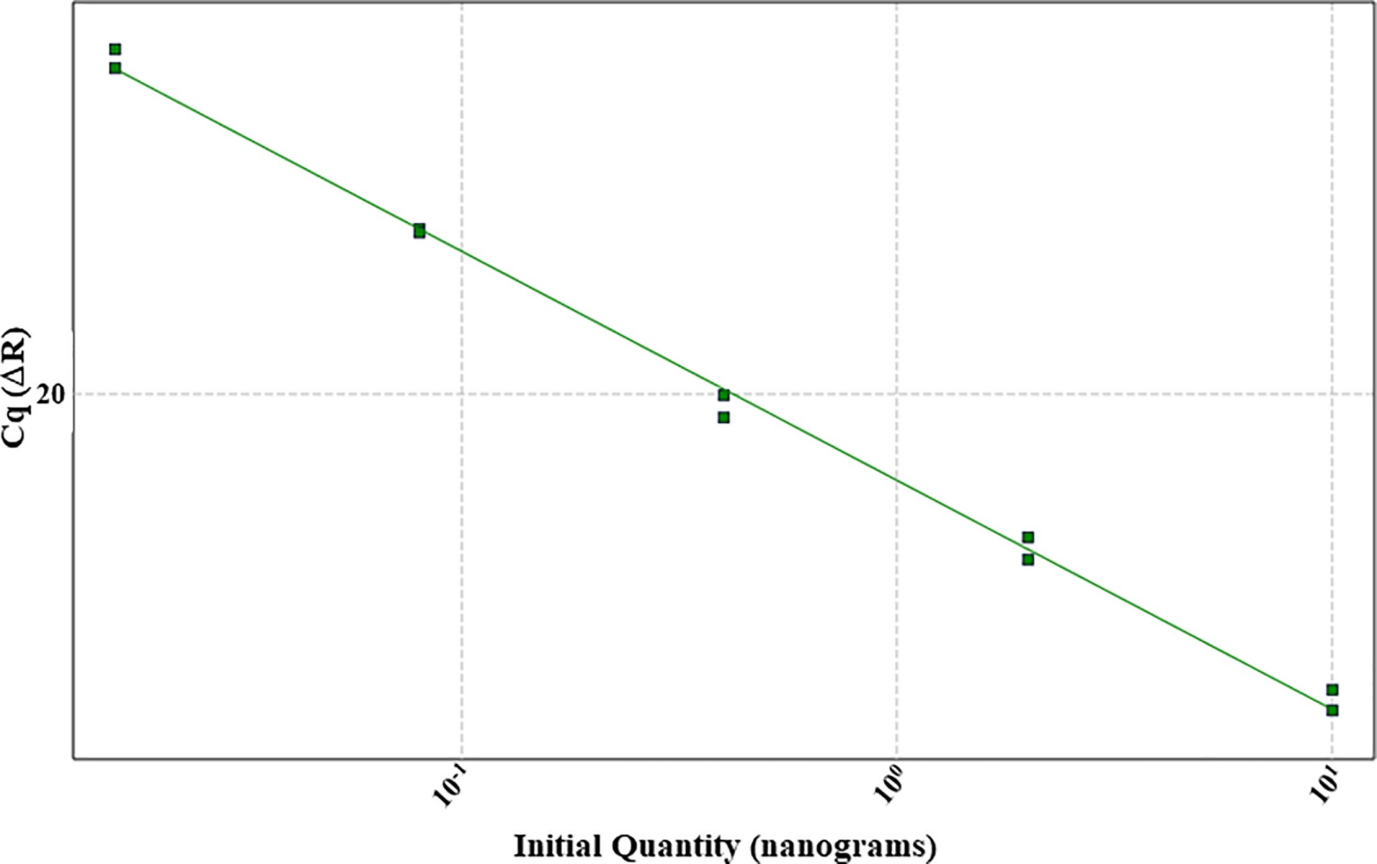
Exemplary standard curve diagram for CLDN10. The graph shows three independent serial dilutions forming linear standard curves in a singleplex RT-qPCR for CLDN10.

**Fig 3:**
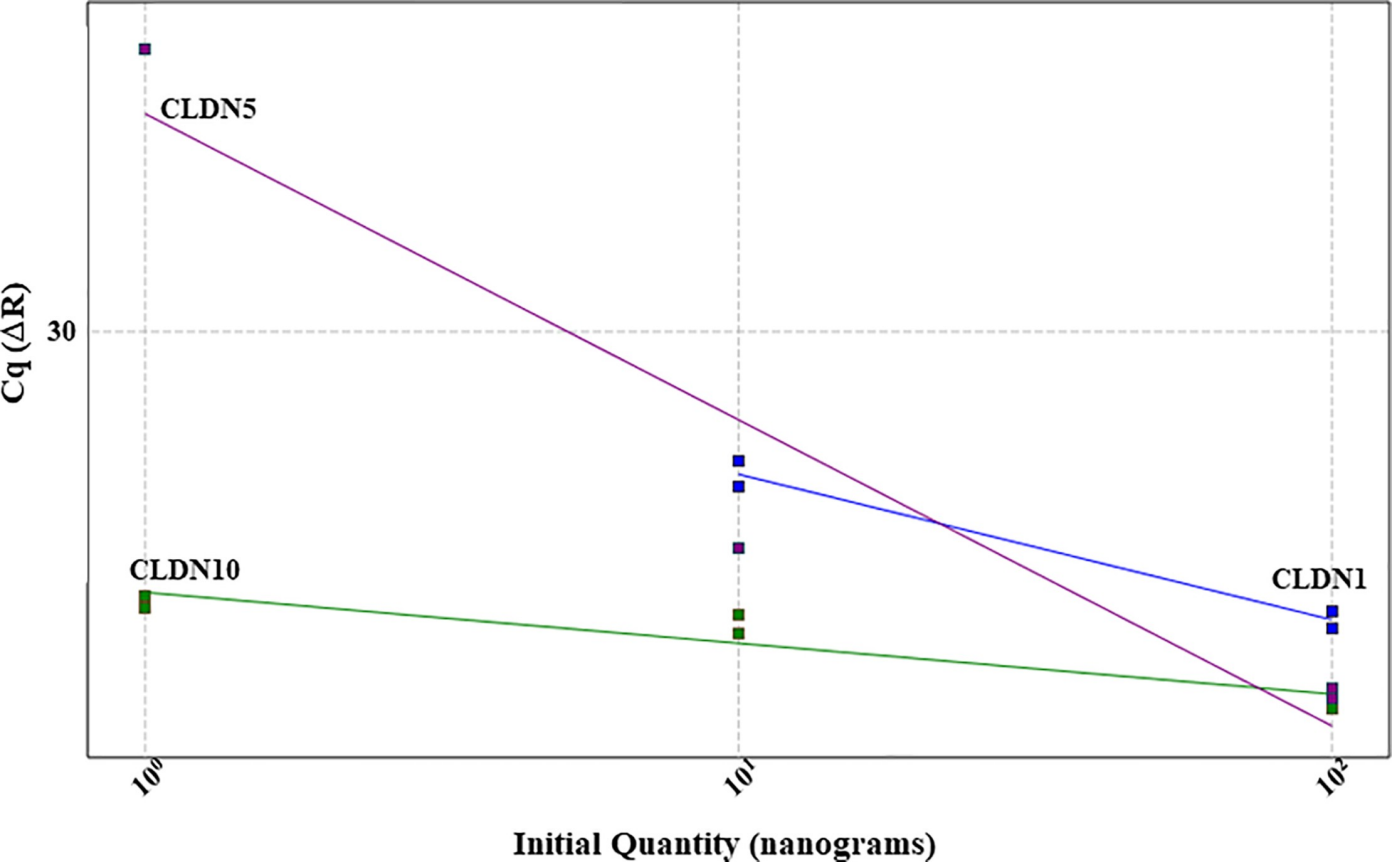
Standard curves of CLDN1, 5 and 10 in a multiplex RT-qPCR assay. Primers and/or probe of CLDN10 are interfering with CLDN1 and 5, indicated by not proportional and not linear standard curves.

**Fig 4:**
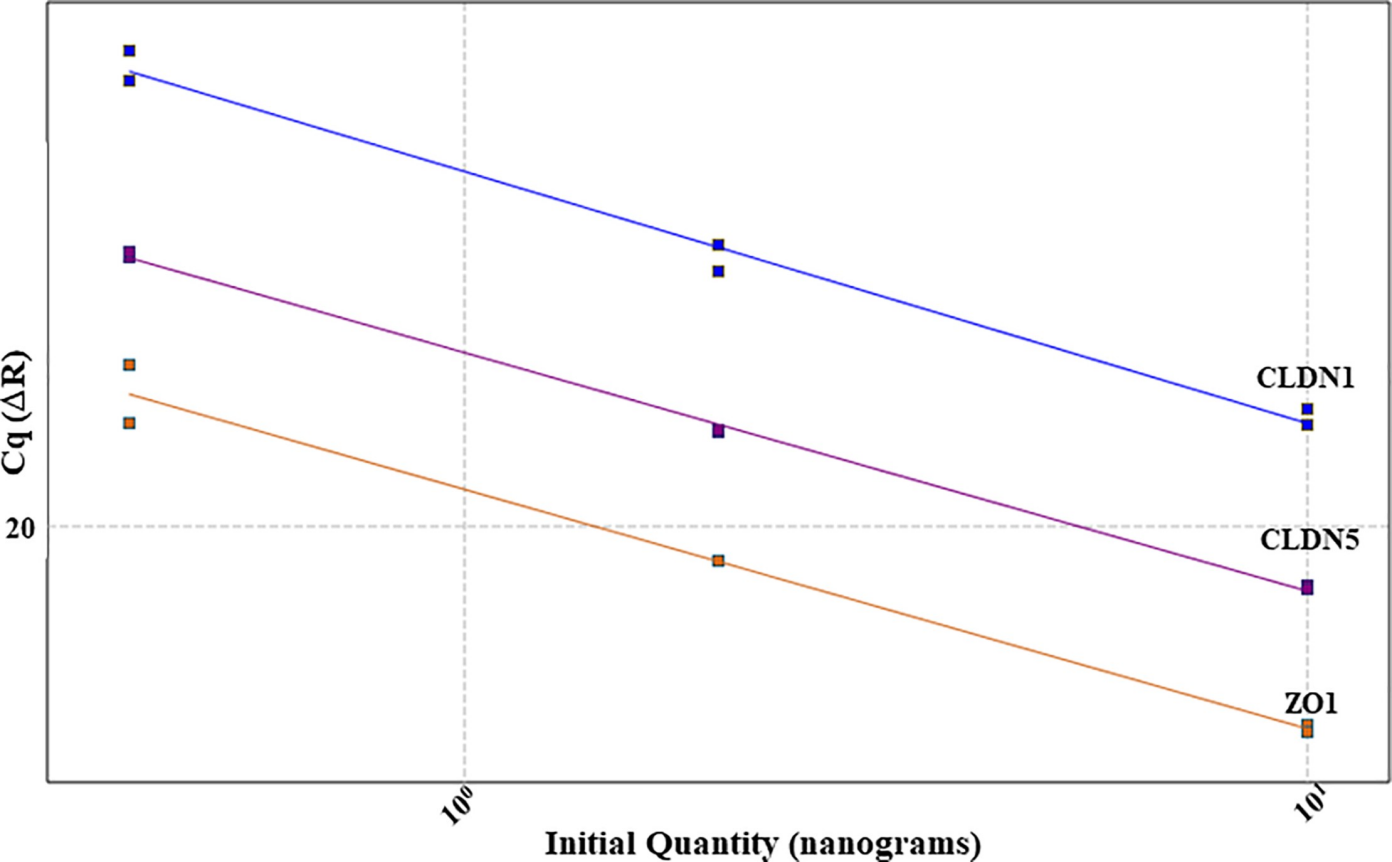
Standard curve of CLDN1, CLDN5 and ZO1. The primers of CLDN1, CLDN5 and ZO1 are forming parallel linear standard curves in a 3x serial dilution enabling accurate quantification of three different target genes in one RT-qPCR run.

Standardization of probe based RT-qPCR was designed individually for jejunum and caecum. To obtain the optimal concentration of primer/probe combination for each selected gene, primer concentrations ranging from 100-600nM and probe from 50-200nM were tested (Table 1). Finally, 100nM concentration was selected for all probes, whereas the concentration of primers varied depending on the gene target (Table 1). For the standardization experiment a 5-fold or 10-fold serial dilution was used to generate the standard curve. Technical variability was controlled by running all sample dilutions in duplicate for each primer/probe combination. During the result analysis, the threshold fluorescence was set low enough to be within the exponential growth and above the background. Additionally, it was confirmed that no background signals were visible above the threshold fluorescence line (Fig 1). Once the threshold was set, three dilutions of the template had to form a linear standard curve (R^2^ = 0.985–1). Together with the slope (-3.1to -3.6), the standard curve was used to determine the quantitation efficiency (Tables 2–5). The efficiency had to reach 100% with a maximal variance of 10%. To confirm that the optimized RT-qPCR protocol is applicable among different animals, three biological replicates and two technical replicates were used for each standardization protocol (Figs 1–4). After standardizing each gene for jejunum and caecum in singleplex (Fig 2), the next aim was to combine as many genes possible in a multiplex system. We managed to incorporate CLDN1, CLDN5 and ZO1 (Fig 3) together in a multiplex system as well as CLDN3, CLDN7 and MD2. A suitable combination couldn’t be found for CLDN10, CLDN19 and OCLN and therefore they remained in singleplex (Fig 4).

### Age-associated changes in tight junction protein expression

Gene expression of the nine TJ genes (ZO1, OCLN, CLDN1, CLDN3, CLDN5, CLDN7, CLDN10, CLDN19, and MD2) was measured from jejunum and caecum of broiler chickens collected at 6 different time points. For normalization, the reference genes 60S ribosomal protein L13 (RPL13) and TATA binding protein (TBP) were run in duplex and verified for the present study [33]. Expression of the reference genes showed very low expression variation in both jejunum (<4.2) and caecum (<1.7) (S1 Fig).

**Fig 5:**
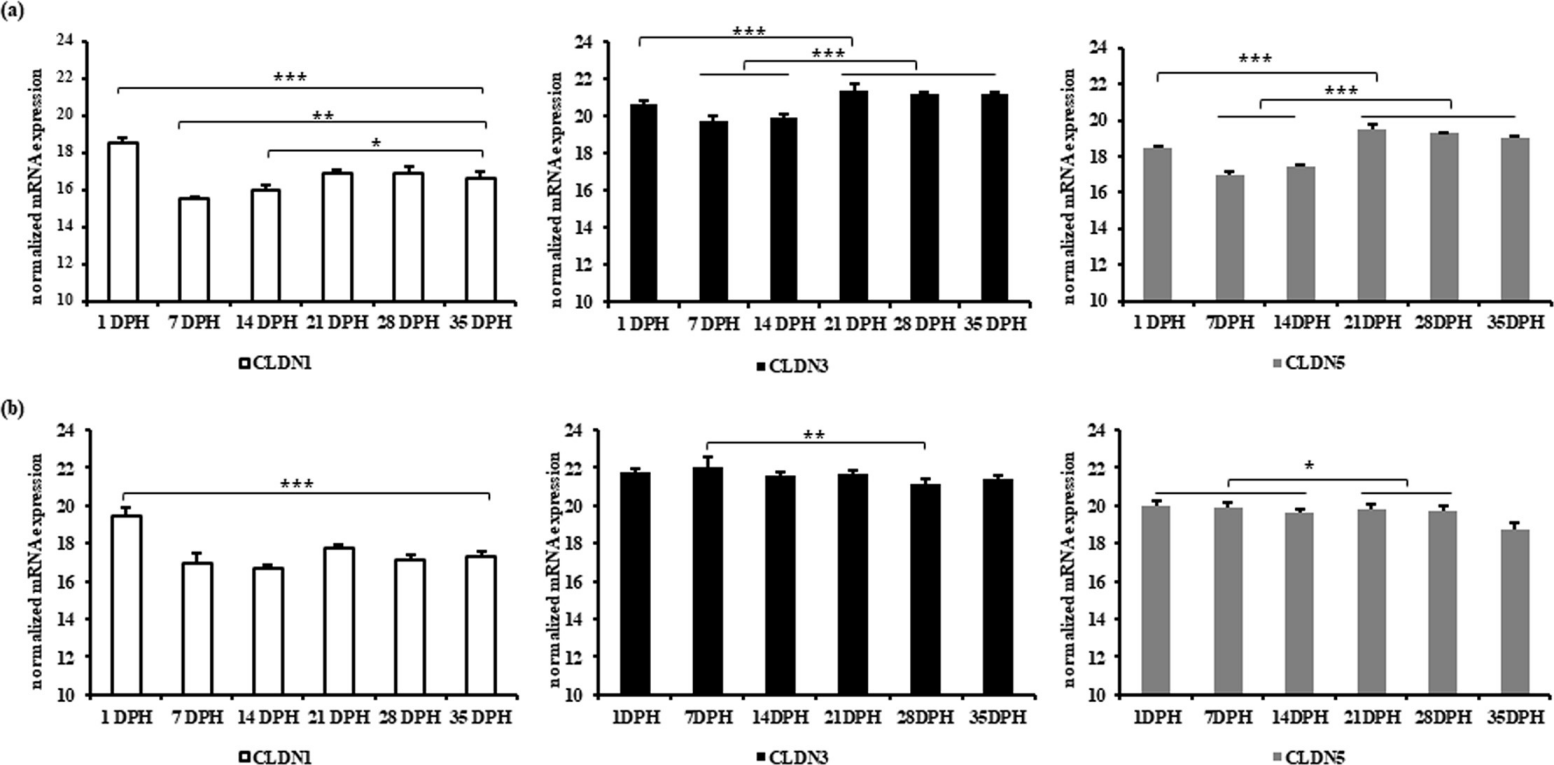
Normalized mRNA expression of barrier-forming claudins in (a) jejunum and (b) caecum at different age of broiler chickens. Results are presented as means with SEM (n = 5 birds). Statistical analysis was performed using ANOVA and Duncan’s test, * = P≤ 0.05, ** = P≤ 0.01, *** = P≤ 0.001. Horizontal lines above the bars indicate no significance.

In jejunum, relative gene expression of barrier TJ proteins, CLDN1, CLDN3, and CLDN5, was significantly decreased (P ≤0.001) at 7 and 14 days post hatching (DPH) as compared to the 1 DPH (Fig 5). However, at 21 DPH, an increase in expression was detected and remained constant thereafter. Similarly, in caecum, CLDN1 was significantly decreased (P ≤0.001) at 7 and 14 DPH as compared to the 1 DPH and increased again thereafter.

**Fig 6:**
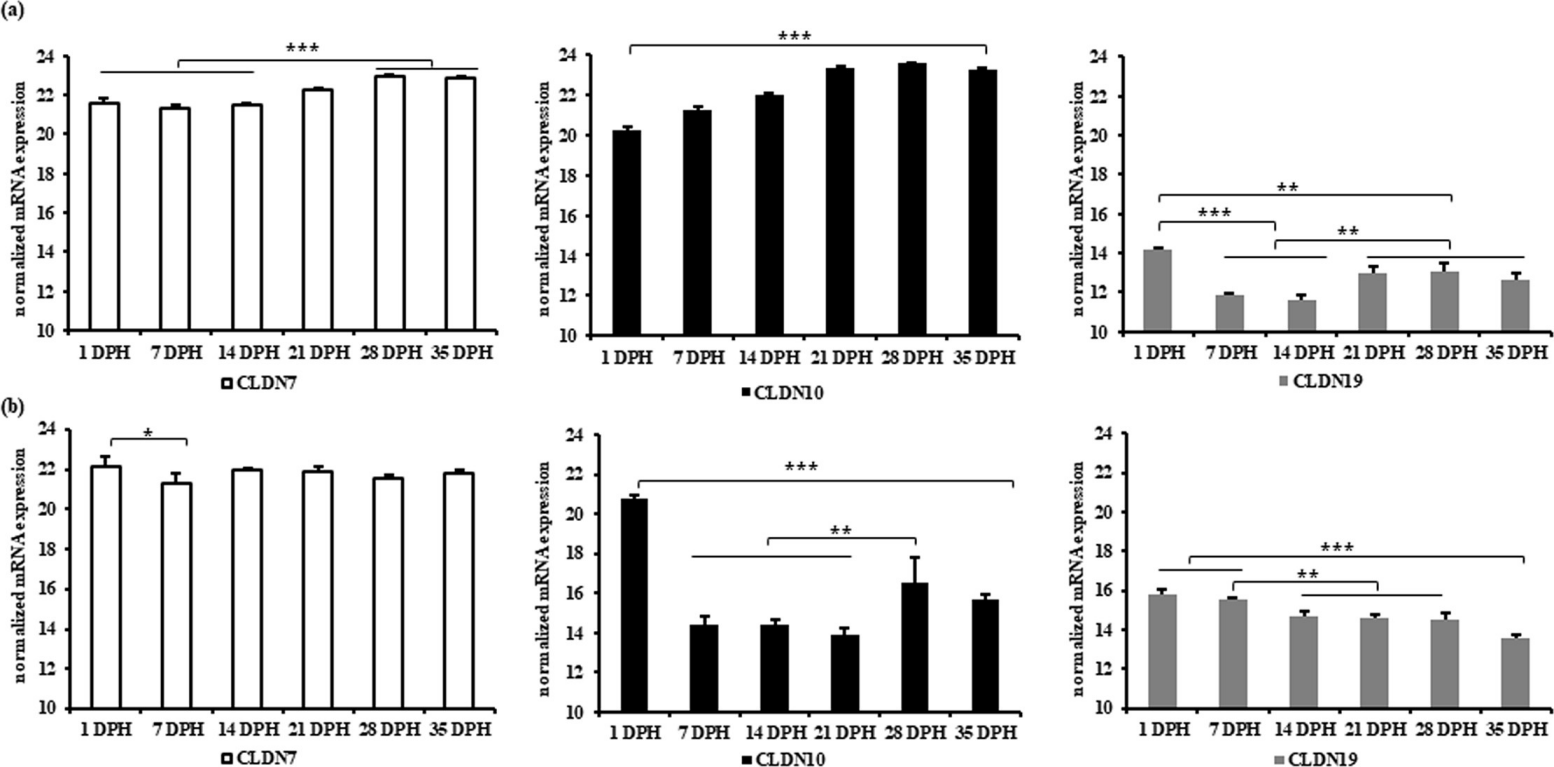
Normalized mRNA expression of pore-forming or ambiguous claudins in (a) jejunum and (b) caecum at different age of broiler chickens. Results are presented as means with SEM (n = 5 birds). Statistical analysis was performed using ANOVA and Duncan’s test, * = P≤ 0.05, ** = P≤ 0.01, *** = P≤ 0.001. Horizontal lines above the bars indicate no significance.

We also found that aging increased the expression of the pore forming TJ proteins, CLDN 7, 10 and 19 in jejunum (Fig 6). However, in caecum, the CLDN7 expression dropped at 7DPH (P ≤0.05) but remained constant thereafter. In contrast to CLDN7, the expression of CLDN10 and CLDN19 was significantly (P ≤0.001) higher at 1 DPH and decreased from 7 DPH onwards.

**Fig 7:**
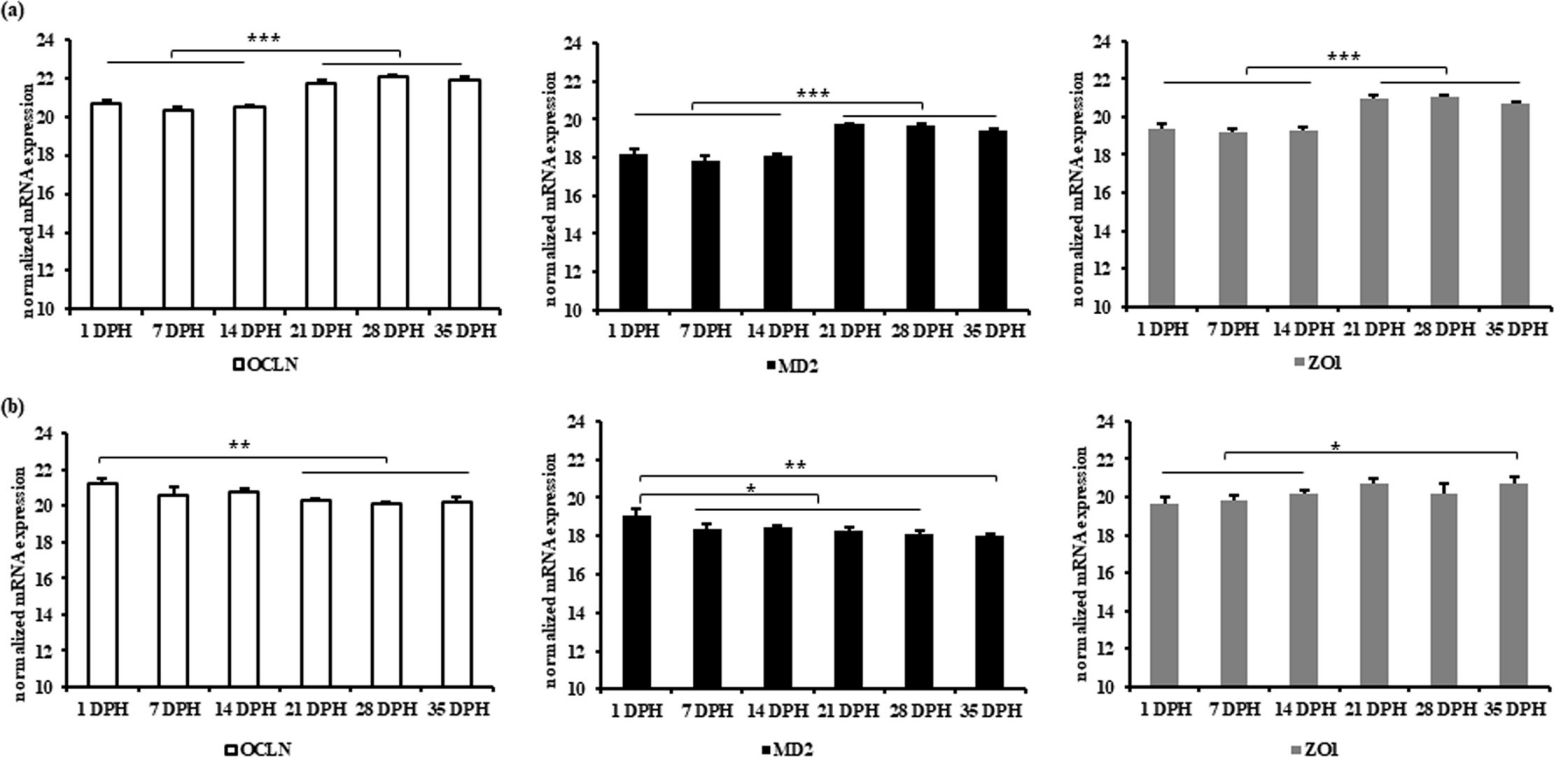
Normalized mRNA expression of OCLN, MD2 and ZO1 in (a) jejunum and (b) caecum at different age of broiler chickens. Results are presented as means with SEM (n = 5 birds). Statistical analysis was performed using ANOVA and Duncan’s test, * = P≤ 0.05, ** = P≤ 0.01, *** = P≤ 0.001. Horizontal lines above the bars indicate no significance.

OCLN, MD2 and ZO1 were expressed with a constant level in jejunum from 1 DPH to 14 DPH followed by an increase in expression from 21 DPH (Fig 7). In caecum, the expression of OCLN and MD2 decreased after 1 DPH, whereas the expression of ZO1 increased from 7 DPH (Fig 7).

## Discussion

Tight junctions play an important role in regulating paracellular ion and nutrient permeation and can affect intestinal absorptive function [11,12]. They play a dominant role in barrier formation, as resolved mainly from studies in mammals. However, the organization of the paracellular barrier of the chicken intestine is still a matter of debate. Despite the high importance of TJ gene expression, there is considerable variation between applied protocols performing RT-qPCR experiments in different laboratories, which are limiting the comparison of results. One of the major current concerns regarding RT-qPCR is data reproducibility and reliability, which is influenced by trial design and data interpretation. In spite of significant improvements in the RT-qPCR assay, there is a considerable divergence in experimental protocols and data analyses from different laboratories with a lack of consistency of proper quality control steps throughout the assay. For instance, error in sample processing by the utilization of wrong reference genes or inappropriate evaluation of the RT-qPCRs overall performance can affect the quality of the results [33,34,36]. Several years ago, the MIQE guidelines (Minimum Information for Publication of Quantitative Real-Time PCR Experiments) have elaborated a framework how to increase the PCR accuracy, however many studies are still not following those guidelines and lacking transparency [34]. Even though such guidelines are largely followed in TJ mammalian research, their implementation for avian TJ, especially the use of an internal probe, has never been reported.

In general, the accurate design of primer and probes increases the assays efficiency and can inhibit non-specific amplification. Designing of primers at exon-exon fitting region on the mRNA, eliminates the detection of genomic DNA and therefore conclusively increases the PCRs’ specificity and reproducibility. Moreover a sequence specific probe design will inhibit potential expression interference with some other genes of the same species [37,38].

Before performing RT-qPCR, RNA quality and quantity assessments are important in order to decrease the possibility of errors in the RT-qPCR analysis, since they can directly affect the Cq values [38,39]. Therefore, in the actual study a detailed evaluation of RNA quantity by NanoDrop and quality by Bioanalyzer were applied for each sample to select samples of high RNA integrity for standardization as well as to accurately dilute RNA quantity to the concentration needed to perform PCR. For all experiments, RIN values ranging from 8–10 were selected to increase the precision and reproducibility.

For optimization of the RT-qPCR quantitation protocol all assays were set up with a TaqMan assay. Choosing a hydrolyzing probe assay is more specific than using SYBR^®^ Green assays, as also the probe is designed for the wanted allele and therefore only hybridizes at the presence of the specific target sequence. However, the standardizing process is more critical. The analytical specificity of probe-based system is greatly increased compared to the SYBR^®^ Green dye. As the non-specific fluorescent dye SYBR^®^ Green binds to any double-stranded DNA, even in the presence of primer dimer formation or with unspecific amplification products, the SYBR fluorescent signal increases correspondingly [31,34].

Another benefit of the probe based RT-qPCR compared to SYBR^®^ Green is the possibility to design a multiplex assay, which can assay several genes simultaneously and by that save time and resources. Implementation of SYBR^®^ Green dye chemistry in multiplex assays is not recommended due to its unspecific binding to any double stranded DNA [40]. Throughout multiplexing the compatibility of the different primer reactions has to be observed because primer dimers can be formed and interfere with the target amplification. Therefore the standard curves have to remain linear and the efficiency mustn’t differ more than 5% by comparing with the singleplex [41,42]. In order to increase the reaction compatibility of each target gene, all PCRs were standardized with the same thermal and amplification cycle. CLDN1, 5 and ZO1 as one group and CLDN3, 7 and MD2 were possible to multiplex. Combining CLDN10, 19 and OCLN didn’t give acceptable results as the reactions seemed to interfere with each other; therefore, those latter assays remained standardized as singleplex for use in the developmental analyses on chicken intestine.

It is well known that age as well as environmental factors do influence the gut barrier in chicken [25,43,44]. In broiler chicken, the commencement of feed intake after hatch promotes intestinal development [24,45]. Especially in the small intestine, epithelial surface is constantly increasing due to villi enlargement and epithelial cell proliferation [46].The present study revealed a highly significant increase in the mRNA expression of the potentially pore-forming claudins 7 and 10 as well as of OCLN, MD2 and ZO1 in jejunum during that period. A precise functional characterization of chicken TJ proteins has not been performed so far. However, it is known from studies in mammals that CLDN10 may form either anion or cation-selective paracellular pores depending on exon inclusion and splicing [47]. One of the proposed functions of the ambiguous CLDN7 is to increase paracellular Na^+^ conductance [48]. Therefore, the increase of structural TJ proteins as well as those, increasing ion permeation across the paracellular space apparently coincide with cell proliferation observed in previous studies and thus appear crucial for the functional development of the small intestine [46,49]. The decreased mRNA expression of the barrier forming claudins (CLDN1, CLDN3 and CLDN5) within the first 2 weeks post hatching and their increase in expression at 21 DPH are likely associated with the reorganization and cellular differentiation for the nutrient uptake and yolk sac regression [46]. Similar results were obtained for CLDN1, CLDN5, ZO1 and OCLN in a recent study of Proszkowiec-Weglarz *et al*. 2020, in which expressional changes of different intestinal developmental markers were observed in the small intestine of broiler chicks fed immediately after hatch or deprived from food for 48h [24].

In caecum, most of the investigated TJ genes showed no or minor changes in mRNA expression after hatch with the noticeable exception of decreases in CLDN1 and in the pore-forming claudins CLDN10 and CLDN19. The main function of the caecum is microbial digestion. It was reported that the caecal microbiota reaches a mass of 10^11^ per gram digesta at the third day after hatching with no significant fluctuations for the next 30 days of life [50]. Bacterial load and fermentation activity within the large intestine will lead to an accumulation of fermentation acids and change of pH. Therefore, it may be speculated that this triggers a decrease in paracellular ion flow, which could be extrapolated from the significant decrease in CLDN10 and CLDN19 mRNA expression within the first or second week after hatching. Interestingly, in mammals CLDN10 expression is mainly located in the ileocaecal region and lowest in the small intestine [51]. Those findings were in disagreement with our findings in the chicken intestine, where mRNA expression of CLDN10 was generally higher in jejunum compared to caecum. We also found that the CLDN19 expression was comparably low in jejunum and caecum, which indicates a limited importance of CLDN19 in the intestinal tract as also noticed in mammals [1,52].

## Conclusion

The constitution of TJs can be used as a marker for gut integrity and understanding the composition of TJ proteins in chickens is crucial to perceive certain physiological and pathological changes in gut TJ barrier. Thus, sequence-specific probes for different TJ genes (claudins 1, 3, 5, 7, 10, 19, zonula occludens 1, occludin and tricellulin) were designed and probe-based RT-qPCRs were established in chickens to possibly monitor gut health and integrity on an mRNA level. Additionally, CLDN1, CLDN5 and ZO1, as well as CLDN3, CLDN7 and MD2 were assayed in multiplex RT-qPCR, minimizing the number of separate reactions and enabling robust testing of many samples. All RT-qPCRs were standardized for chicken jejunum and caecum samples underlining the potential and applicability to other organ tissues. Furthermore, age-associated changes of mRNA expression in the chicken intestinal TJ barrier were resolved which could help to demonstrate how compartmental separation and transepithelial transport takes place at different ages. Simultaneously, it points to differential regulation in paracellular ion permeability in chicken small and large intestine after hatch. These findings enhance our understanding on how restructuring of TJ proteins may alter the susceptibility to age-related gut disorders. A well-functioning epithelial barrier is crucial to inhibit bacterial translocation across the host intestinal epithelium to the inner organs. The paracellular barrier of the chicken intestine is still not resolved but our RT-qPCR results contribute in selecting the most crucial TJ proteins at certain time points for further quantitative analysis. In future studies it would therefore be important to focus on the localization and quantification of those proteins in the intestinal tissue to gain a deeper understanding of the molecular mechanisms underpinning pathophysiological changes based upon various aetiologies.

## Supporting information

S1 Fig Cq value fluctuations of references genes RPL13 and TBP in jejunum and caecum of chickens during age development. The fluctuations of Cq value in jejunum was 4.11 for RPL13 and 2.01 for TBP and for caecum 1.69 for RPL13 and 1.9 for TBP.
(TIF)
