# Supplementary figures and images for: Establishment of a novel probe-based RT-qPCR approach for detection and quantification of tight junctions reveals age-related changes in the gut barriers of broiler chickens
Source: PLoS One. 2021 Mar 5;16(3):e0248165. doi: 10.1371/journal.pone.0248165 (PMC7935255; doi:10.1371/journal.pone.0248165)

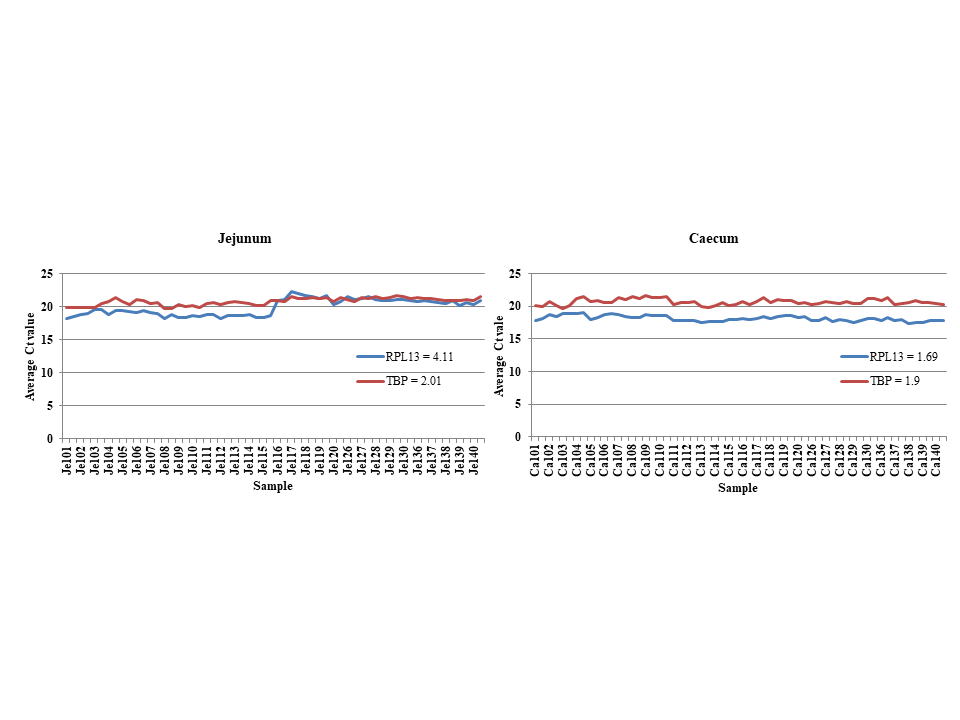

Supplement: S1 Fig — The fluctuations of Cq value in jejunum was 4.11 for RPL13 and 2.01 for TBP and for caecum 1.69 for RPL13 and 1.9 for TBP. (TIF) [file pone.0248165.s001.tif]
